# Relationship between arsenic skin lesions and the age of natural menopause

**DOI:** 10.1186/1471-2458-14-419

**Published:** 2014-05-02

**Authors:** Fakir Md Yunus, Musarrat Jabeen Rahman, Md Zahidul Alam, Samar Kumar Hore, Mahfuzar Rahman

**Affiliations:** 1 BRAC Research and Evaluation Division, BRAC Centre, 75 Mohakhali, Dhaka 1212, Bangladesh; 2 ICDDR, B – Water, Sanitation and Hygiene Research Group, Center for Communicable Diseases (CCD), Moyeen Center, House-9b, Road-3, Gulshan-1, Dhaka 1212, Bangladesh; 3 Department of Biology, University of Texas at Arlington, 76010 Arlington, TX, USA; 4 ICDDR, B- Chronic Disease Epidemiology and Genetics Research Group, Centre for Control of Chronic Diseases (CCCD), 68 Shahid Tajuddin Ahmed Sharani, Mohakhali, Dhaka 1212, Bangladesh; 5 ICDDR, B – Public Health Sciences Division, the Centre for Health and Population Research, 68 Shahid Tajuddin Ahmed Sharani, Mohakhali, Dhaka 1212, Bangladesh; 6 UChicago Research Bangladesh, House 4, Road 2B, Sector 4, Uttara, Dhaka, Bangladesh

**Keywords:** Arsenic skin lesions, Arsenic exposure, Age of Menopause, Reproductive period, Epidemiology

## Abstract

**Background:**

Chronic exposure to arsenic is associated with neoplastic, cardiovascular, endocrine, neuro-developmental disorders and can have an adverse effect on women’s reproductive health outcomes. This study examined the relationship between arsenic skin lesions (a hallmark sign of chronic arsenic poisoning) and age of natural menopause (final menopausal period) in populations with high levels of arsenic exposure in Bangladesh.

**Methods:**

We compared menopausal age in two groups of women – with and without arsenic skin lesions; and presence of arsenic skin lesions was used as an indicator for chronic arsenic exposure. In a cross-sectional study, a total of 210 participants were randomly identified from two ongoing studies— participants with arsenic skin lesions were identified from an ongoing clinical trial and participants with no arsenic skin lesions were identified from an ongoing cohort study. Mean age of menopause between these two groups were calculated and compared. Multivariable linear regression was used to estimate the relationship between the status of the arsenic skin lesions and age of natural menopause in women.

**Results:**

Women with arsenic skin lesions were 1.5 years younger (p <0.001) at the time of menopause compared to those without arsenic skin lesions. After adjusting with contraceptive use, body mass index, urinary arsenic level and family history of premature menopause, the difference between the groups’ age at menopause was 2.1 years earlier (p <0.001) for respondents with arsenic skin lesions.

**Conclusions:**

The study showed a statistically significant association between chronic exposure to arsenic and age at menopause. Heavily exposed women experienced menopause two years earlier than those with lower or no exposure.

## Background

Chronic inorganic arsenic poisoning has been characterized by cutaneous abnormalities which includes melanosis (hyperpigmentation) and keratosis [1]. Bangladesh retained WHO’s previous guideline value for arsenic concentration in drinking water as a national standard which suggests 50 μg/L or lesser amount as safe [2], although the current WHO guideline value suggests it as 10 μg/L or less [3]. However, in Bangladesh, out of a total population of 164 million, around 22 million people are drinking water with arsenic concentration >50 μg/L, and, of them, 5.6 million people are drinking water with arsenic concentrations even > 200 μg/L [4]. Ground water arsenic contamination is the largest mass poisoning in the history of Bangladesh [5]. Several researchers found that chronic exposure to high concentrations of arsenic causes both carcinogens and non-carcinogenic effects [6-9]. This effect includes arsenical skin lesions i.e. melanosis and keratosis, vascular diseases, liver and neurotoxicity, chronic cough, diabetes mellitus, adverse pregnancy outcomes, and impaired child development. Arsenic exposure elevates risks of cancer, which persists even decades after exposure has ceased [10].

Previous studies reported the effect of chronic arsenic exposure on reproductive health outcomes in women and noted significant association with adverse pregnancy outcomes such as spontaneous abortion, stillbirth and preterm births. Arsenic toxicity may also increases risks of birth defects [11-14]. Other study identified chronic arsenic exposure a female reproductive toxicant, and as an endocrine disruptor, therefore, considered as a major threat for female reproductive health. In animal (rat) experiments, arsenic toxicity deprives oestrogen receptors [15] resulting in interrupted normal activity of organs such as uterine lining, skin, breast tissue, etc. that are responsible for menopause. Oestrogen is responsible for the onset of menstruation in adolescents; however gradual deprivation of receptor eventually results early menopause [16]. In relation to this, another study suggested that arsenic causes delayed menarche [17].

Considering both human and animal studies, this study hypothesized that chronic arsenic exposure characterized by the presence of arsenic skin lesions could have a relationship with the natural menopausal age. This may mean that arsenic associated with early or delayed menopause - in either way, this could be an undesirable outcome. If menopause occurs early, there will be a reduction in reproductive period that means women would likely to experience of cardiovascular diseases, neurological diseases, osteoporosis and psychiatric diseases earlier in their lives [18]. On the other hand, delayed menopause may increase the risk of breast and ovarian cancers due to their prolonged exposure to oestrogen [19].

## Methods

### Study area and population

The study was conducted in Laksam upazilla of Comilla district, and Araihazar upazilla of Narayangonj district in Bangladesh. Participants with arsenic skin lesions were selected from Laksam upzilla as this area is highly contaminated to arsenic [20]. Participants without arsenic skin lesions were selected from Araihazar, another arsenic contaminated area. Though we collected participants from two different areas, socio-demographic characteristics were similar as both are located in the eastern part of Bangladesh.

Participants with arsenic skin lesions were selected randomly using the database from an ongoing randomized clinical trial "Bangladesh Vit-E and Selenium Trial (BEST)" (ClinicalTrails.gov:NCT00392561) conducted in Laksam, Comilla. This clinical trial aims to determine the supplementation of vitamin E, selenium or their combination that could have beneficial effects on arsenic induced skin cancer. It is a double blind, randomized, placebo-controlled 2 × 2 factorial clinical trial conducted among 7,000 individuals with pre-malignant skin lesions for the prevention of cancers and deaths in Bangladesh. This study enrolled both male and female participants with arsenic skin lesions i.e. melanosis and keratosis within the age limit of 25–65 years. In a recent publication using the baseline data of this clinical trial, associations was found between arsenical skin lesions with common chronic diseases such as hypertension, diabetes, asthma, and peptic ulcer disease [21]. While participants with non-arsenic skin lesions were selected randomly from the ongoing cohort database. This prospective cohort study "Health Effects of Arsenic Longitudinal Study (HEALS)" aims to investigate the intermediate and long-term pre-malignant, malignant and non-malignant health effects of among ~30,000 men and women who were naturally exposed with arsenic through drinking water. This cohort enrolled both male and female participants with a history of drinking water at least three years from the same tube-well within the age limit of 18–75 years. Mean urinary arsenic concentrations of male (140 μg/L) and female (136 μg/L) participants were found in the baseline survey [22]. Both of these databases enrolled participants in 2006 and 2007. Finally, participants for this study were randomly selected based on two basic criteria- one was women with a presence or absence of arsenic skin lesions, and another was women, who had experienced menopause within the last five years (from 2011) with the age limit of 35–55 years.

Considering 80% power, 5% level of significance, Unexposed/Exposed ratio of 1, percent of unexposed with outcome 30 and percent of exposed with outcome 50 using the "OpenEpi" version 2.2 software (Developer: Emory University, Rollins School of Public Health) the sample size was calculated [23]. This yielded a total number of 190 participants, among them 95 were exposed and 95 were unexposed to arsenic. Finally a total 210 eligible women were interviewed to collect relevant information using the "recall" method. Among them, 105 participants had arsenic skin lesions and 105 participants did not. Using the range of eligibility from 35 to 51 years yielded a total of 187 participants for further analysis out of total interviewed 210 participants. Among the selected participants, 95 had arsenic skin lesions and 92 had no arsenic skin lesions.

### Data collecting procedure

Detail menopausal history and socio-demographic information was collected using close-ended structured questionnaires consisting of easy-to-understand questions with appropriate response options. The researchers developed the questionnaire and pretested in a neighbouring village similar to the study area.

Ten trained female staffs were recruited for this study as interviewers. They conducted a door-to-door survey to identify the potential participants from the randomly selected list of those databases. All staff were from the study areas and had a very good cultural understanding of the participants. Moreover, interviewers were kept blinded to the objectives of the study. Questionnaires were read and comprehensively explained in front of the potential participants, since they voluntarily accepted to participate in the study, they were then asked to sign or put a thumbprint on the written informed consent paper. For both groups, interviewers back-checked the presence or absence of arsenical skin lesions i.e. melanosis, leucomelanosis and keratosis by full body examination under sunlight. Before the final enrollment into the study, screening was done on under certain criterions such as current menstruation, menopause occurred in less than 1 year (in order to get the final menstrual period) or menopause occurred in more than 5 years, history of using depot injection in last five years, conducted total abdominal hysterectomy (TAH), bilateral salpingo-oophorectomy and severe medical conditions. Finally, women were asked to recall their menopausal age. It was assumed that they could recall accurately as it an important life event for women.

### Exposure assessment

Arsenic exposure was determined through the presence or absence of arsenic skin lesions. This is because skin lesions are a hallmark sign of chronic arsenic poisoning— hyperkeratosis and melanosis are considered as an indicator of high arsenic exposure [24,25] whereas urinary arsenic level indicates recent exposure within a few days of the specimen collection [26].

### Outcome assessment

Natural menopause was determined according to the World Health Organization (WHO) definition "Permanent cessation of menstruation resulting from the loss of ovarian follicular activity" which corresponds to a single point in time- the final menstrual period (FMP) [27]. This study used the final menstrual period as an outcome variable.

### Covariate and other variables

Several studies suggest that a number of factors may affect natural menopausal age, such as smoking, educational attainment, income, marital status, employment, parity, contraceptive use, family history of premature menopause, cancer and history of chemotherapy or radiotherapy and body mass index [18,28-35]. Among these, a few factors such as smoking, parity, marital status, income, use of contraceptive methods, body mass index and family history of premature menopause prevailed among the respondents. Socio-demographic information such as number of children, education, marital status, monthly household income was collected. Other than these, detailed menstrual information such as age of menarche, age of menopause, menstrual regularity and its frequency, use of contraceptives, and family history of premature menopause were also collected. To obtain the family history of premature menopause, participants were asked whether their mother or sister had experienced menopause before the age of 40 years [28]. Information related to arsenic such as duration of exposure to arsenic and urinary arsenic level were taken from two databases of clinical trial and cohort study in the year of 2006–2007. Other factors that may affect the age of menopause such as smoking history and body mass index (BMI) were reviewed retrospectively. BMI was calculated using the standard method (weight in kg/ height in meter^2^) [36]. The reason behind reviewing information from the mentioned database (2006–2007) was to explore other factors at the time of menopause.

### Statistical analysis

Both descriptive and inferential analysis was done by using SPSS software- version 17 (Chicago, IL), and Microsoft Excel (2010). Frequency tables and summary statistics were done to check the missing data, out of range values and to assess distributions of continuous variables, and logic checks were done. Independent-sample t tests were undertaken to compare the mean difference of age of menarche, age of menopause and reproductive period between participants with and without arsenic skin lesions. Reproductive period was calculated by deducting the age of menarche from the age of menopause.

Multivariable linear regressions were done to estimate the relationship between arsenic skin lesions and age of menopause after adjusting for potential confounding variables. Initially univariate analyses such as chi-square tests were done to explore the association between the two groups of participants, their socio-demographic characteristics such as age, occupation, monthly household income, education, marital status, menstrual regularity and frequency (that they experienced in the past) and with the potential factors that affect the age of menopause. These factors, such as smoking, family history of premature menopause, number of children, BMI, use of contraceptives and urinary arsenic level (μg/g creatinine) were included in the initial model. In the final regression model, only significant covariates were included. Categorical variables were entered in the final model as dummy variables.

### Ethical review

The study protocol and recruitment procedure (access to the databases) were approved by the Ethical Review Board of the James P Grant School of Public health, BRAC University as per the existing rules.

## Results

Participants background, detail menopausal information and association between groups are presented in Table 1. Mean age (±SD) of the respondents was 48.0 (±3.6) years. Mean age (±SD) of menarche, menopause and reproductive age was 14.00 ± 1.36, 44.95 ± 3.35 and 30.94 ± 3.51 respectively. The outcome variable ‘age of menopause’ was normally distributed. Most of the participants were housewife 184 (98.4%). Mean (median) of monthly household income was 9019(8000) BDT (Bangladeshi taka) [116 (103) US$]. The majority of women 114 (61%) had no formal education, 68 (36.4%) women had completed primary education and only 5 (2.7%) completed secondary education. Most (165) were married (88.2%). In terms of menstrual regulation and frequency, most (168) of the participants (89.8%) had experience of regular menstruation (i.e. once a month). Out of total (95) arsenic skin lesions participants, most of them 91(48.7%) had arsenic skin lesions for more than 10 years.

**Table 1:** Distribution of socio-demographic characteristics and menstrual details of the study population by arsenic skin lesions

**Variables**	**N**	**%**	**Arsenic skin lesions**			
			**Yes (N = 95)**	**No (N = 92)**	**Mean difference**	**p**
**Age** (years); Mean ± SD	48.00 ± 3.64	-	47.14 ± 3.48	48.89 ± 3.60	1.74	.001
**Occupation**						
Housewife	184	98.4	93	91	-	.22
Daily Labour	2	1.1	2	0		
Govt Service	1	0.5	0	1		
**Monthly household**	9019 (8000) BDT	-	9476 (7000) BDT	8549 (4000) BDT	927 BDT	.28
**Income** (BDT, US$); Mean (median)	116 (102) US$		122 (90) US$	110 (51) US$	12 US$	
**Education**						
No formal education	114	61.0	53	61	-	.07
Primary completed	68	36.4	41	27		
Secondary completed	5	2.7	1	4		
**Marital status**						
Single	2	1.1	2	0	-	.51
Married	165	88.2	83	82		
Widowed	16	8.6	8	8		
Separated	3	1.6	2	1		
Divorced	1	0.5	0	1		
**Menstrual regularity**						
Yes	168	89.8	86	82	-	.81
No	19	10.2	9	10		
**Menstrual Frequency**						
Twice a month	2	1.1	1	1	-	.30
Once a month	169	90.4	88	81		
Once in every 2 months	10	5.3	3	7		
Once in every 3 months	4	2.1	3	1		
Once in >3 months	2	1.1	0	2		
**Arsenic skin lesions duration**						
<10 years	4	2.1	4	0	-	-
>10 years	91	48.7	91	0		
**Age of Menarche**; Mean ± SD	14.00 ± 1.36	-	14.17 ± 1.37	13.82 ± 1.33	.355	.069
**Age of Menopause**; Mean ± SD	44.95 ± 3.35	-	44.17 ± 3.28	45.74 ± 3.25	1.568	.001
**Reproductive period**; Mean ± SD	30.94 ± 3.51	-	29.99 ± 3.31	31.91 ± 3.48	1.914	.001

*Abbreviation: SD* Standard Deviation.

Among those characteristics, age (p = 0.001) was found significantly lower in with arsenic skin lesions group (47.1 ± 3.5) in comparison to without arsenic skin lesions groups (48.9 ± 3.6). Education (p = 0.07), marital status (p = 0.51), monthly household income (p = 0.28), menstrual regularity (p = 0.81) and frequency (p = 0.30) were not significantly different between groups. Age of menarche was similar (p = 0.06) between two groups, however the menopausal age (p <0.001) and reproductive period (p <0.001) were significantly different between the groups. The result indicates 3.5 months delayed age of menarche and 1.5 years early menopause occurred in arsenic skin lesions participants compared to participants without arsenic skin lesions. Approximately, 1.9 years decrease in reproductive period (p = 0.001) occurred among participants with arsenic skin lesions (29.99 ± 3.31 years) compared to participants without arsenic skin lesions (31.91 ± 3.48 years).

Distribution of potential factors such as smoking, family history of premature menopause, number of children, use of contraceptive methods, urinary arsenic level, and BMI between two groups of participants are presented in Table 2. Participant’s number of children was normally distributed and mean (±SD) was 4.74 ± 1.79. Of the factors, family history of premature menopause (p = 0.001) and use of contraceptives (p = 0.01) were found to be positively associated between the groups. Continuous variables such as body mass index (BMI) were found significantly lower (p = .03) among women with arsenic skin lesions (18.93 ± 3.03) in comparison to women without arsenic skin lesions (19.94 ± 3.48). However, urinary arsenic levels were found significantly higher (p = 0.001) among individuals with arsenic skin lesions (328.01 ± 284.17) in comparison to individuals without arsenic skin lesions (156 ± 193.00).

**Table 2:** Factors that affect menopausal age

	**Arsenic skin lesions**		**p**
	**Yes (N = 95)**	**No (N = 92)**	
**Smoking**			
Previous	1	0	1.00
Never	94	92	
**Family history of premature menopause**			
Yes	17	1	.001
No	43	31	
Don’t know	35	60	
**Number of Children**; Mean ± SD	4.74 ± 1.79	4.91 ± 1.79	.52
**Contraceptive Used**			
Yes	47	62	.01
No	48	30	
**Body Mass Index** (BMI)	18.93 ± 3.03	19.94 ± 3.48	.03
**Urinary As Level** (μg/g creatinine)	328.01 ± 284.17	156 ± 193	.001

A multivariable linear regression model was undertaken to adjust all the potential confounders are presented in Table 3. An overall 2.1 years decrease in menopausal age was observed (p < 0.001) in women who had arsenic skin lesions (44.17 ± 3.28) compared to women without arsenic skin lesions (45.74 ± 3.25) after adjusting contraceptive use, urinary arsenic level, body mass index and family history of premature menopause.

**Table 3:** Relationship between arsenic skin lesions and age of menopause

**Model**	**Coefficients**^**a**^	**p**	**CI 95%**	
			**Lower**	**Upper**
Contraceptive Use Y/N	-.531	.286	-1.510	.449
Arsenic Skin Lesions Y/N	-2.102	.001	-3.207	-.996
Body Mass Index (BMI)	.002	.977	-.145	.150
Urinary Arsenic Level (μg/g creatinine)	.002	.075	.000	.004
Family history of premature menopause (Dummy1- yes)	.321	.717	-1.425	2.067
Family history of premature menopause (Dummy2-Don’t know)	-.296	.571	-1.326	.734

^a^Outcome Variable: Age of Menopause.

## Discussion

We assessed the relationship of chronic arsenic exposure (marked by the arsenic skin lesions) and the natural menopausal age in rural Bangladesh. An overall 3.5 months of delay was observed on the menarcheal age (p = 0.06) among the participants. Even though this was not significant, the direction of the difference observed is consistent with other study [17], who assessed the effect of arsenic on the menarcheal age, and noted a significant delayed onset of menarcheal age among adolescents exposed to arsenic by drinking tube well water in West Bengal, India. We did not have reliable data on their arsenical skin lesions at the time of their menarche, however, in the context of Bangladesh, it can be easily assume that all the participants had been drinking the same arsenic-contaminated water their whole lives as they had never moved from their community since birth. Consistent consumption of the contaminated water gradually led to arsenic poisoning.

Secondly, our finding revealed that chronic arsenic exposure seems to have a relationship with the menopausal age. Comparing the mean age of menopause between two groups (with or without arsenic skin lesions), an overall 1.5 years (p = 0.001) of earlier menopause was observed among the women who had arsenic skin lesions compared to those who did not have arsenic skin lesions. Mean reproductive period was 1.9 years lower among the participants with arsenic skin lesions compared to the participants without arsenic skin lesions. The finding was also similar in the multivariable linear regression analysis. This result suggests that chronic exposure to arsenic, as marked by the presence of skin lesions is associated with early menopause. That means holding all other potential confounders constant, women with arsenic skin lesions would experience 2.1 years early menopause than the women without arsenic skin lesions. Therefore, these women are at higher risk of developing non-communicable diseases, including cardiovascular diseases, neurological diseases, osteoporosis and psychiatric diseases earlier in their lives compared to the women without arsenic skin lesions [18]. On the other hand, this result also indicates that women would be at lower risk of breast and ovarian cancers due to less duration of exposure to oestrogen [19,37,38]. The current study is, therefore, important because it sheds light on the relationship of high and long-term arsenic exposure on the age of menopause.

It is not clear how the arsenic metabolism and toxicity process influences the age of natural menopause in human. In rat experiments, arsenic has been identified as a reproductive toxicant and endocrine disruptor, and has been observed to impact the structure and the functions of the uterus in female rat [15]. In humans, arsenic interacts with steroids, particularly oestrogen that may lead to adverse health effects in women. Oestrogen receptors are present in various organs such as uterine lining, skin, breast tissue, bone and blood vessels. If any of these organs are deprived of the substrate, menstrual disorders may result [39]. Arsenic also may cross placental barriers and exposure to this toxicant may occur when the foetus is still within the mother’s womb, although its effects may visibly appear much later in life [37,39].

In this study, all the participants with or without arsenic skin lesions have high urinary arsenic level, but the arsenic skin lesions group showed significantly (p = .001) higher mean level (328.01 ± 284.17) than their counterpart (156 ± 193.00). Although the urinary arsenic level is consider the most reliable method for measuring arsenic exposure, it is particularly sensitive to recent exposure— within a few days of the specimen collection [26]. Therefore, in this study, we deemed skin lesions as the main criteria for determining arsenic exposure because skin lesions indicates long-term exposure and this long-term arsenic exposure is more important to consider for certain health conditions than recent exposure. Arsenic skin lesions is also considered as the hallmark sign of chronic arsenic poisoning and as an indicator of high arsenic exposure [24,25].

An important strength of this study was the direct measurement of arsenic skin lesions in each individual. Another positive issue was the adjustment for relevant early menopausal risk factors among the study women such as number of children, family history of premature menopause, use of contraceptives, urinary arsenic level and BMI.As participants (with or without arsenic skin lesions) were selected randomly, selection bias was less likely to occur. One potential limitation of the study is using the self-reported data on the age of menopause and family history of premature menopause, therefore, there is a risk of recall bias through differential misclassification of natural age of menopause among women with arsenic lesions. To avoid differential misclassification of outcomes, we blinded all study staff that includes interviewers and participants assessing for both exposures to outcome and outcome to exposures status. Along with this, we conducted training among the interviewers in order to adhere to the question, and answer the format strictly, with the same degree of questioning on objective measures (hard data) for both with or without arsenic skin lesions participants to reduce interviewer bias. However, we assume that any fluctuation of recalling menopausal age and the family history of premature menopause is likely to affect equally in participants with or without arsenic skin lesions. Another limitation of this study is that bio-markers for arsenic exposure such as hair, nail and blood concentration of arsenic were not measured. As this is a cross-sectional study, temporality between arsenic exposure and menopause cannot be claimed. Prospective cohort study with such population could elucidate this relationship. Although we recruited participants from two different places, as a proxy for socioeconomic status, monthly household income was not significantly different (p = 0.35) among arsenic skin lesions (90 US$)) and without arsenic skin lesions (103 US$) participants.

## Conclusions

This study suggests that natural menopausal age has relationship with chronic arsenic exposure (marked by the presence of arsenic skin lesions). The study found menopause occurs earlier when women had arsenic skin lesions. This early experience of menopause results in women having an increased risk of suffering from serious diseases such as cardiovascular, neurological, osteoporosis, and psychiatric diseases. This study also reveals that women with arsenic skin lesions experienced shorter reproductive period in their lifetime. Moreover, evidence connecting high and long-term arsenic exposure to altered age of menopause has strengthened the argument that arsenic directly affects reproductive health. Further research, especially with a larger sample, could elucidate the interaction between arsenic exposure, oestrogen hormone, and the menopausal age. To the best of our knowledge, we did not find any other study that carried out on the relationship between arsenic skin lesions and the age of menopause.

## Competing interests

All authors declared that they have no competing interests.

## Authors’ contributions

FMY contributed to the all stages of research that includes conception of the research, proposal writing, data analysis and manuscript writing. MJR, and MZA contributed to data analysis and manuscript writing. SKH and MR contributed to the conception of the research, data analysis and reviewing manuscript. All authors read and approved the final manuscript.

## Authors’ information

Fakir Md Yunus: Formerly at James P Grant School of Public Health, BRAC University, 68 Shaheed Tajuddin Ahmed Sarani Mohakhali, Dhaka-1212, Bangladesh.

## Pre-publication history

The pre-publication history for this paper can be accessed here:

http://www.biomedcentral.com/1471-2458/14/419/prepub
